# Some Observations on the Development of Superior Photocatalytic Systems for Application to Water Purification by the “Adsorb and Shuttle” or the Interphase Charge Transfer Mechanisms

**DOI:** 10.3390/molecules191219557

**Published:** 2014-11-26

**Authors:** Cooper Langford, Maryam Izadifard, Emad Radwan, Gopal Achari

**Affiliations:** 1 Department of Chemistry, University of Calgary, 2500 University Dr. NW, Calgary, AB T2N 1N4, Canada; 2 Department of Chemistry University of Calgary, 2500 University Dr. NW, Calgary, AB T2N 1N4, Canada; E-Mail: izadifam@ucalgary.ca; 3 Department of Water Pollution Research, National Research Centre, Cairo 12311, Egypt; E-Mail: emadk80@gmail.com; 4 Department of Civil Engineering, University of Calgary, 2500 University Dr. NW, Calgary, AB T2N 1N4, Canada; E-Mail; gachari@ucalgary.ca

**Keywords:** TiO_2_, photocatalysis, adsorb and shuttle, charge transfer, electron storage, carbon, zeolite, WO_3_

## Abstract

Adsorb and shuttle (A/S) and interfacial charge transfer are the two major strategies for overcoming recombination in photocatalysis in this era of nanoparticle composites. Their relationships are considered here. A review of key literature is accompanied by a presentation of three new experiments within the overall aim of assessing the relation of these strategies. The cases presented include: A/S by a high silica zeolite/TiO_2_ composite, charge transfer (CT) between phases in a TiO_2_/WO_3_ composite and both A/S and CT by composites of TiO_2_ with powered activated carbon (AC) and single-walled carbon nanotubes (SWCNT). The opportunities presented by the two strategies for moving toward photocatalysts that could support applications for the removal of contaminants from drinking water or that lead to a practical adsorbent for organics that could be regenerated photocatalytically link this discussion to ongoing research here.

## 1. Introduction

At present, the vast majority of the commercial application of photocatalysis depends on passive processes that may accomplish their photochemical goals slowly. This is a consequence of the small quantum yields typically reported and the limited UV energy available from low free or the low cost sources, either sunlight or fluorescent lamps installed for lighting purposes. Earlier in development of photocatalysis, major effort was expended on modification of the structure of TiO_2_ to overcome one or both of the limitations. So far, radical improvement has eluded researchers. In recent years, improvement in manipulating the chemistry of the nanoscale has fueled a fresh campaign to achieve improvement by creating hybrids of the photocatalyst with other phases, such that the field of applications might expand. Two key strategies can be found in the literature. One is the combination of a phase with TiO_2_ that can act as an acceptor for either the conduction band electron or the valence band hole to substantially inhibit recombination. The other is the “adsorb and shuttle” strategy that combines a phase that more extensively adsorbs the substrate and delivers it by surface diffusion to an adjacent TiO_2_ site. Using an adsorbent, which could be photocatalytically generated, is advantageous for drinking water and waste water photocatalytic treatment systems. These two strategies emerged before the nanoparticle outburst, and some fundamental aspects from earlier studies will be discussed below along with more recent key references. Some new results will be reported. The transfer of carriers between phases with electron transfer from TiO_2_ to WO_3_ is an area related to the water program. The two oxides have little difference in adsorption, making this nearly a “pure” charge transfer case. Results for a pure adsorb and shuttle case will come from a study of zeolite ZSM-5 on TiO_2_, a candidate in the effort to produce adsorbents that are photocatalytically regenerated. Finally, some results for the mixed case of carbon and TiO_2_ will be discussed.

### 1.1. Adsorb and Shuttle (A/S)

The key issues with A/S were elucidated in a series of studies from Yoneyama’s laboratories in the late 1990s. The approach involved loading a large excess of TiO_2_ onto particles of well-known adsorbents. In the case of the substrate, 3,5-dichloro-*N*-(3-methyl-1-butyn-3-yl)benzamide (propyzamide), photodegradation was studied using photocatalyst coated onto clays, zeolites and activated carbon [1]. The catalysts were all 70 wt % TiO_2_. Adsorption isotherms fitted the Langmuir model. The Langmuir maximum adsorption capacity parameter was critical, but so was the specific initial extent of adsorption after dark equilibration, a measure of adsorption “strength”. Apparently, the decomposition rate (measured as the sum of solution and adsorbed loss) of propyzamide was large in the order of naked TiO_2_ > 70 wt % TiO_2_/mordenite > 70 wt % TiO_2_/SiO_2_ > 70 wt % TiO_2_/AC (activated carbon), the order being opposite of the order of the Langmuir adsorption constant (strength), except for SiO_2_. This appears to suggest that adsorb and shuttle was not useful. However, the rate of CO_2_ production (mineralization, the goal of most work) was in the order: 70 wt % TiO_2_/AC > 70 wt % TiO_2_/SiO_2_ > 70 wt % TiO_2_/mordenite > naked TiO_2_. This is exactly the order of the initial adsorbate loading of the photocatalysts at initiation of irradiation. The important following point is that tracking of intermediates showed that most were in solution in the case of bare TiO_2_, but the overwhelming majority of intermediates was adsorbed in the case of TiO_2_/AC. Thus, adsorb and shuttle can be a powerful tool for retaining intermediates and the completion of mineralization.

The key point of adsorb and shuttle, A/S, is that the concentration of the substrate close to the TiO_2_ surface should greatly increase the probability of meeting between the substrate and a photoactivated site. An elementary illustration of this is the increase of a quantum yield when a favourable substrate concentration is raised, as is the case of an increase of quantum yield from 0.001 to 0.15 as the propanol concentration is raised from 0.001 M to 0.05 M [2]. Moreover, it is known that interfacial redox can compete well with recombination. For example, Colombo and Bowman [3] provide evidence that electron transfer can compete with recombination on a femtosecond time scale for particles encountering. Still, for the adsorbed substrate to reach the active site, it must migrate by surface diffusion. Consequently, surface diffusion must not be slow. Using propionaldehyde as a substrate, the relative surface diffusion was estimated on films containing TiO_2_ by following the consumption of all of the substrate on a film illuminated over a part of the area. Takeda *et al*. [4] found relative surface diffusion to be in the order TiO_2_/mordenite > TiO_2_/silica > TiO_2_/alumina > TiO_2_/AC > TiO_2_/zeolite A. An interesting related example of the problem of surface diffusion arose in an effort to produce a practical adsorbent based on a ZSM-5 hydrophobic zeolite absorber decorated with TiO_2_ to allow photocatalytic regeneration, a project that reached the pilot scale. Vaisman *et al*. [5] reported that as the adsorption capacity increased, the photocatalytic kinetics decreased proportionately.

In summary, effective adsorb and shuttle, A/S, requires adsorbents with a delicate balance of adequate adsorption capacity, sufficient absorption strength and facile surface mobility. As the systems described above suggest, the best case seems to be a large excess of TiO_2_ over the adsorbent.

### 1.2. Interphase Charge Transfer 

The classic example of the reduction of the recombination rate is the deposition of small quantities of Pt on the TiO_2_ surface to capture electrons in the Pt phase. The literature is extensive. There are also a number of semiconductor oxides that could inhibit recombination in TiO_2_ if the matching of band gaps permits the transfer of an electron from the TiO_2_ conduction band to a lower band edge or permits the hole in the TiO_2_ conduction band to migrate to a higher energy conduction band edge. The examples are WO_3_, which can play the role of electron acceptor and Ni(OH)_2_, which can function as a hole acceptor. If the separation is to be long lived and build up in the acceptor phase, there must be electrochemical compensation. For example, if WO_3_ accepts electrons, we look to W(V) chemical traps requiring cationic compensation. Tatsuma *et al*. [6] demonstrated that the presence of ionic conduction through water could support long-lived energy storage by supplying H^+^ as the cation (note that the TiO_2_ hole reacting with water produces a compensating H^+^). Oxygen discharge of the “stored” electrons in WO_3_ is slow, so energy storage providing reactivity in the dark is feasible. Thus, increased hole reactions from reduced recombination can be coupled to energy storage. Subsequently, Tatsuma *et al*. [7] demonstrated the bactericidal effect of the stored energy.

### 1.3. Carbon

Carbon has been mentioned above (along with a zeolite, silica or clay) as an adsorbent. It is, of course, the most widely used adsorbent for organics. However, there is also significant direct evidence of excited state electron transfer from TiO_2_ to carbon. A striking example is the puzzle presented by Kedem *et al*. [8], who reported enhanced stability in polymer nanofibres containing TiO_2_ and carbon nanotubes (CNTs). The CNTs reduced the rate of photocatalytic degradation of the (polyacrylonitrile) matrix. However, this did not interfere with photocatalytic reactions with several organic substrates and even enhanced the degradation of rhodamine 6G dye. This set of conflicting results can be understood as the CNTs functioning to transport charge carriers from the TiO_2_ to CNT sites, which may induce a variety of effects (e.g., superoxide formation) depending on the degradation mechanism and reaction loci.

In combinations of carbon with photocatalysts, we must ask: what is the relative role of charge transfer *vs*. adsorb and shuttle?

The 6G dye measurements, made on aliquots of solution not accounting for the dye adsorbed on the CNTs, raises a final basic point. Xu and Langford [9] made an important observation about strongly adsorbed substrates using the dye XB3. For all the reactions induced by UV or visible light, the apparent initial rate of X3B loss in the aqueous phase increased with the initial equilibrated concentration of X3B. However, when the rate was determined by the decreased concentration both in the aqueous phase and on the catalyst surface, increase of real initial rate with the initial equilibrated concentration was observed only in the visible-light-induced reaction. That is, only when the reaction was initiated by dye sensitization that initiates dye oxidation directly upon excitation. It is important to measure total degradation, solution and surface, as done in the work in Yoneyama’s group cited above.

## 2. Results and Discussion

### 2.1. TiO_2_/ZSM-5 Adsorb and Shuttle

The chosen adsorbent is the high silica zeolite ZSM-5 in a configuration similar to the system of Vaisman *et al*. [5] designed as an adsorbent for photocatalytic regeneration. ZSM-5, with a highly hydrophobic surface, presents an adsorption profile resembling activated carbon, but weaker for some highly hydrophobic molecules and stronger for more polar organics. It is used here in composite with Degussa P25 TiO_2_ (PZS). The percentages of three different model substrates adsorbed on the surface of PZS and P25 after 30 min of stirring in the dark are shown in Table 1. It can be seen that there is an unequivocal increase in the amount of the 2,4,6-trichlorophenol (2,4,6-TCP) substrate adsorbed on the surface of PZS with a drastic increase observed in the case of sulfamethoxazole (SMX), while for atrazine, the adsorption was the least. The overall order of increase in percent adsorption is SMX > 2,4,6-TCP > atrazine.

**Table 1:** Adsorption percentage of the different model compounds at initial concentrations of 50.0 mg/L on the surface of PZS and P25 after the dark period. SMX, sulfamethoxazole; 2,4,6-TCP, 2,4,6-trichlorophenol.

Compound	Photocatalyst	
	Degussa P25%	PZS%
SMX	2.60	18.00
2,4,6-TCP	29.20	41.90
Atrazine	2.40	5.64

**Figure 1:**
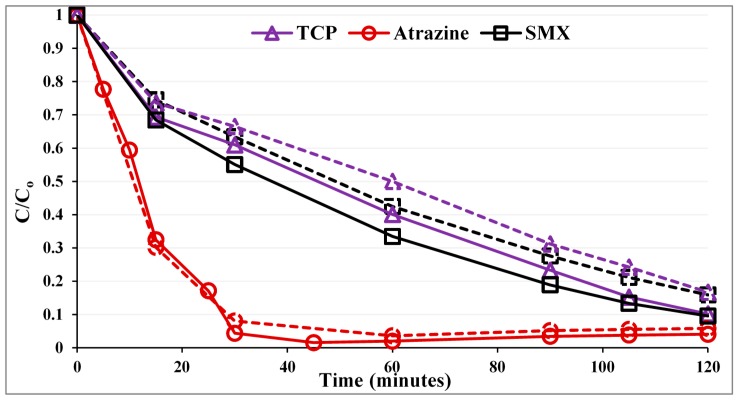
Photocatalytic degradation of the substrates with a UVA LED photoreactor. Solid lines, PZS (P25:ZSM-5:silica gel = 0.3:0.5:0.5 g/L); dashed lines, Degussa P25 (0.3 g/L).

Figure 1 compares the photocatalytic performance of the PZS to that of commercial Degussa P25 as a reference. The first point corresponds to the normalized concentration after the dark adsorption period. Pseudo first-order rate constants (k) along with correlation coefficients (R^2^) are listed in Table 2 (because of the rapid reaction, it was not a certain that the atrazine data do fit first order kinetics).

**Table 2:** Pseudo first order rate constants (min^−1^) and R^2^ fit measure for the substrates.

Compound	Degussa P25 min^−1^		PZS min^−1^	
SMX	k = 0.0147	R^2^ = 0.996	k = 0.0189	R^2^ = 0.9965
2,4,6-TCP	k = 0.0137	R^2^ = 0.977	k = 0.0179	R^2^ = 0.982
Atrazine	k = 0.055	R^2^ = 0.923	k = 0.0976	R^2^ = 0.966

As can be seen in Figure 1, for SMX and 2,4,6-TCP, the PZS shows noticeably higher photocatalytic activity than the Degussa P25. The enhancement in efficiency is not in proportion, as the adsorption strength of these compounds on the zeolite surface is different. While for atrazine, the photocatalytic activity was within the error, the same for both Degussa P25 and PZS. The order of photocatalytic activity improvement is SMX ~ 2,4,6-TCP > atrazine, which is consistent with the order of improved adsorption. This suggests that adsorb and shuttle may have been effective.

**Figure 2:**
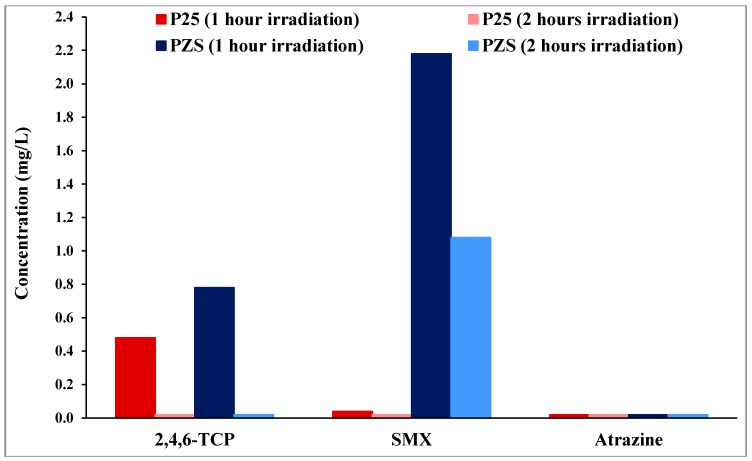
Residue results of the substrates on the surface of PZS and Degussa P25 after one and two hour irradiation. The left bar shows 1-h data and the (clearly visible only for SMX) right bar 2-h data.

Substrates were extracted to track the extent of the retention of the initial substrate on the adsorbent surface during reaction. The amounts are reported in units of the solution concentration equivalent. Figure 2 shows data for 60 and 120 min.

2,4,6-TCP residue on the surface of both photocatalysts after 1 h of irradiation was small (less than 1 mg/L solution equivalent compared to an initial total of 50 mg/L). After 2 h of irradiation, no 2,4,6-TCP residues were detected on the surface of either photocatalyst. Furthermore, it can be seen from Figure 2 that, when the initial extent of dark adsorption is small, no residues were detected on the surface of either photocatalyst, as seen for atrazine on both photocatalysts and even for SMX on P25. The residue of SMX on the surface of PZS after 1 h of irradiation was 2.2 mg/L, which reduced to 1 mg/L after 2 h of irradiation. The comparison of the initial extent of dark adsorption results (Table 1) with these results suggests that 2,4,6-TCP surface diffusion [4] is faster than SMX.

**Figure 3:**
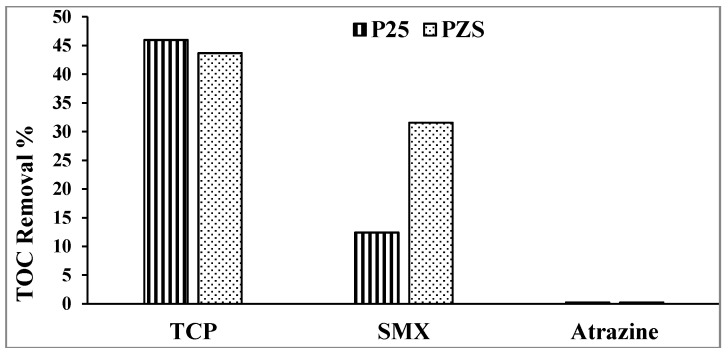
Percentage TOC removal after 1 h of irradiation.

The key advantage of A/S is the promotion of mineralization [1]. The percentage of TOC removal of the substrates using PZS and Degussa P25 after 1 h of irradiation is shown in Figure 3. Using PZS improves the mineralization of SMX and shows that 2,4,6-TCP is ~45% mineralized after ~50% loss in the substrate. No mineralization of atrazine is seen, in agreement with McMurray *et al*. [10].

The results are a reminder that atrazine is one of the rare compounds not mineralized on TiO_2_. The reaction stops at cyanuric acid [10].

### 2.2. Interphase Charge Transfer: TiO_2_/WO_3_

**Figure 4:**
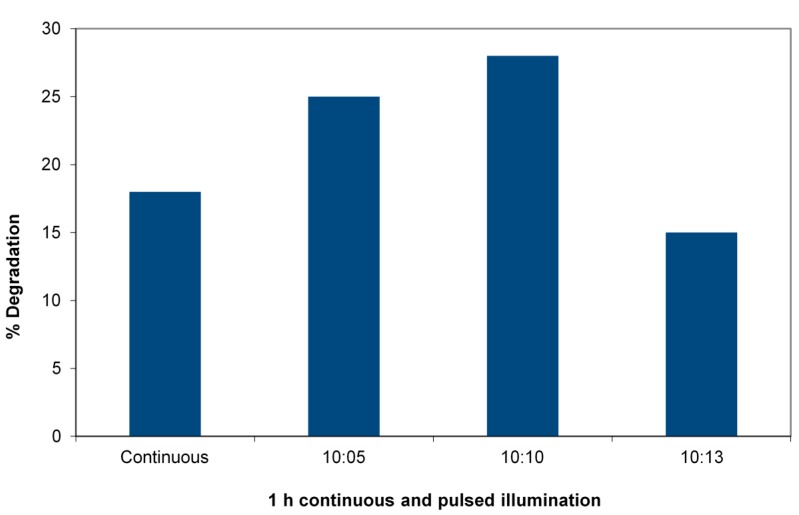
Pulsed *vs*. continuous irradiation in a 365-nm LED reactor degrading 4-chlorophenol (4-CP). Charge is 0.05 g WO_3_/TiO_2_ and 5 mL of 33 mg/L 4-CP receiving (4.3 ± 0.2) × 10^16^ photons/s. Total irradiation: 60 min in each case, duty cycle (ratio minute light:minute dark) varied.

**Figure 5:**
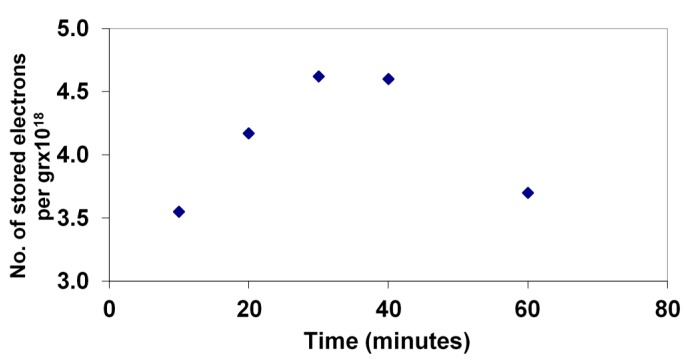
Stored electron levels during the photocatalytic degradation of 4-CP measured by Fe(III) titration with Fe(II) detection by phenanthroline complexation.

One of the most striking demonstrations of the reduction of recombination rates arises from the persistence of reactivity after irradiation is terminated [11]. In this experiment, 4-chlorophenol (4-CP) was chosen as a substrate to avoid any A/S contribution. Figure 4 shows the percent degradation of 4-CP after one hour of irradiation in a slurry of TiO_2_/WO_3_ in a 33-ppm solution of the 4-CP using the 365-nm LED reactor to allow precise pulsing. The duty cycle is varied. The figure shows the striking case of the efficiency increase in degradation reactions of 4-chlorophenol. A 1:1 duty cycle at a constant total dose with a pulse time of 10 minutes (10^4^ ms) increases the efficiency of energy utilization by almost a factor of two. Titration of available electrons after termination of illumination by Fe(III) reduction is shown in Figure 5.

The extent of electron storage during the photodegradation of 4-CP was monitored by rapid titration with Fe(III) of a sample of the solution after successive times of illumination. Figure 5 shows the levels during the degradation of the 33 mg/L sample, as described above.

It has been reported [7] that charge storage with WO_3_ allows inhibition of *E. coli* that are exposed in the dark to TiO_2_/WO_3_. This is confirmed by data in Table 3, where illumination was UVA (365 nm), and the sample was outflow water from a secondary wastewater treatment plant. This water contains a mix of other organic molecules that can compete for reaction with excited TiO_2_/WO_3_. A 50-mL waste water sample with 10-ppm fulvic acid (FA) solution (as a supplemental hole scavenger) + 0.33 g WO_3_-TiO_2_ was used. In a first experiment, 50 mL of contaminated water was added to the TiO_2_ slurry, and the mixture was incubated for 5 h. In the subsequent experiment, after 30 min in the dark, the slurry was irradiated for 2 h in the 365-nm LED reactor. Post irradiation, the 50-mL contaminated water sample was added and incubated for 5 h. The samples were measured for coliforms by the Colilert tray method, widely used for water monitoring. Note that the initial sample counts are not reproducible due to the biological activity in the stock.

**Table 3:** Coliform counts after exposure to TiO_2_/WO_3_ without and with pre-charging for 2 h. (MPN = most probable number of colonies).

Sample	Total Coliform (MPN)	*E coli* (MPN)	Decrease in *E. coli*
Stock solution	1,921.2	246.8
WO_3_/TiO_2_ (dark blank)	872.0	161.0	35%
Stock solution	1,732.9	488.4
WO_3_/TiO_2 _charged	108.4	26.6	95%

### 2.3. Carbon

**Figure 6:**
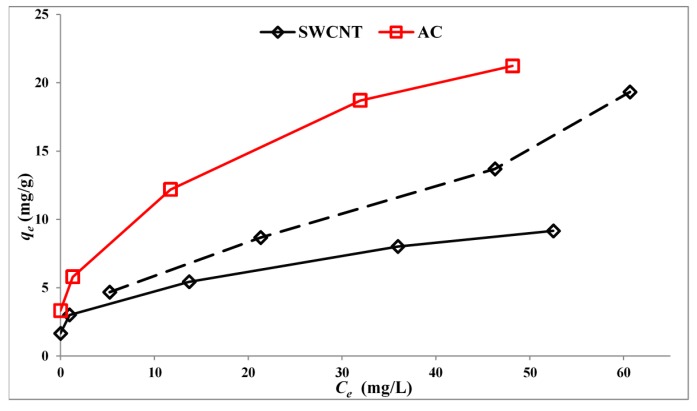
Adsorption isotherms at 22 °C for SMX on activated carbon (AC) or single-walled carbon nanotubes (SWCNT). SWCNT: solid line, 5.0% by weight; dashed line, 0.25% by weight. C_e_ = solution concentration; q_e_ = adsorbed weight per unit mass (mg/g).

Carbon is a good adsorbent and can act as an electron acceptor [12]. Consequently, both mechanisms may arise. An interesting potential application is the removal of emerging contaminants from water. For this reason, composites were made using the well-established TiO_2_, Degussa P25 reference point. Figure 6 shows the adsorption isotherms for SMX on P25 loaded with 5% by weight of either AC or SWCNTs or 0.25% by weight of SWCNTs. These values are chosen, because preliminary results show good rates for photocatalysts with 0.25% SWCNTs and 5% activated carbon (AC).

The comparative amounts adsorbed as a function of the loading of carbon on TiO_2_ is interesting and offers a possible explanation for the preliminary observation that 5.0% AC and 0.25% SWCNTs were both favourable loadings for the study of the photocatalytic reaction. The amounts of SMX adsorbed are similar.

**Figure 7:**
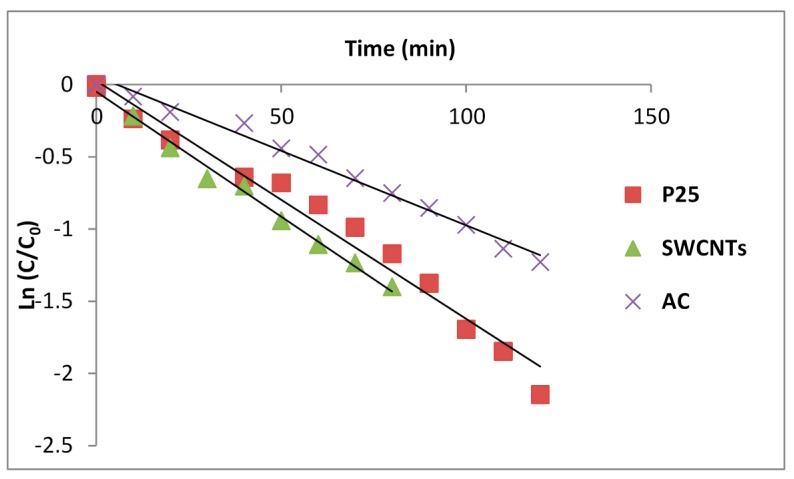
Kinetics of the total loss of SMX on irradiation of slurries in a 365-nm batch reactor.

A series of representative results for SMX degradation on the three photocatalysts are collected below for a photocatalyst loading of 1.0 g/L and initial solution SMX concentrations of 2.4 × 10^−4^ M. After the dark adsorption equilibration period, solution concentrations were 1.1 × 10^−4^ M for P25/AC, 2.3 × 10^−4^ M for P25 and 1.4 × 10^−4^ M for P25/SWCNT. Table 4 collects the rate constants. The rate constants for the total loss of substrate are compared in the table to apparent “rate constants” calculated from solution concentrations alone. Where adsorption is small (P25), the solution value is in agreement. However, differences are quite significant when carbon is added. The data shown here are a reminder that many rate constants in the literature have been reported on solution data where adsorption may have been significant. This can be quite misleading. It is especially true for studies involving dye molecules [9]. Figure 7 shows the kinetics of total SMX loss with P25, P25/AC and P25/SWCNT as photocatalysts plotted as first order reactions. Powdered activated carbon appears to act as an inhibitor in these circumstances. SWCNTs may be marginally accelerating. This is consistent with reports that surface diffusion is faster on SWCNTs than powdered activated carbon [8].

**Table 4:** First order rate constants for the total loss of SMX (4.2 × 10^−4^ M) with three photocatalysts.

Photocatalyst	Rate Constant (min^−1^)	R^2^ Fitting Parameter	Apparent Solution “Rate Const.” (min^−1^)
P25/AC	0.0103	0.988	0.0154
P25	0.0165	0.980	0.0166
P25/SWCNT	0. 0173	0. 990	0.0227

In an effort to detect possible electron storage from charge transfer in TiO_2_/SWCNTs composites, reactions were carried out in the pulse irradiation mode with pulse cycle times down to 100 ms, with duty cycles of 50%, 30% and 10%. (with the relaxation time of TiO_2_ alone near 70 ms. [13]l a shorter pulse time would not distinguish carbon effects). In no case was a significant difference between pulse irradiation and continuous irradiation observed for a constant energy dose. This could suggest that the SWCNTs function only in the A/S mode, but the alternative explanation, given the direct evidence for electron transfer, is that carbon mediates rapid electron transfer to O_2_. There is evidence that nanocarbon can function as an efficient conductor, delivering the electrons [8] to an acceptor. Yao *et al*. [12] reported results for a physical mixture of carbon nanotubes with TiO_2_ nanotubes. The solution conversion rates were superior to TiO_2_ alone, but the physical mixture rate was only about half of the composite rate. The positive effect in a physical mixture was taken to be evidence of electron transfer. It does seem unlikely that the time of an encounter would allow efficient shuttle action, unless encounters are “sticky”. Yao *et al*. used a dispersant to minimize this. An experiment with a physical mixture of P25 with SWCNTs (ratio as above) started from 95 mg/L SMX, which led to a surface concentration equivalent to 24 mg/L after dark adsorption (clearly carbon loading). Four hours of irradiation reduced the solution concentration and the surface residual to values comparable to those for the composite, tending to support a role for electron transfer.

### 2.4. Discussion

#### 2.4.1. Adsorb and Shuttle (A/S)

The new photocatalyst is assembled in a silica matrix. This resembles the systems described in Vaisman *et al*. [5] that were used to construct an adsorbent to be regenerated photocatalytically. Since good results are achieved with an excess of adsorbent over TiO_2_, the adsorbent that is regenerated may be a good A/S target. The kinetic behaviour for the decrease of three substrates is reported in Figure 1. The TOC data in Figure 3 underlines the key advantage of A/S. Intermediates are retained on the photocatalyst assembly, and mineralization is favoured as observed by Torimoto *et al*. (1996). In the regeneration application, mineralization is important and may lead to the choice of a poorer catalyst for the loss of the initial target compound. The key challenge to the use of A/S is that adsorb and shuttle systems are highly dependent on the substrate.

#### 2.4.2. Interphase Charge Transfer

A system to unambiguously demonstrate the transfer of carriers that inhibit recombination needs to minimize the potential for A/S. WO_3_ may be a better adsorbent for some organic substrates than TiO_2_, but the difference is not as great as with ZSM-5 or carbons. To further minimize A/S, a reaction with a weakly adsorbed substrate can be chosen. In this case, 4-chlorophenol is the chosen substrate. The pulse experiment is the most striking demonstration of the reduction of recombination, since it requires that carriers may remain to react after the light goes off. TiO_2_ itself will have a short relaxation time for a reaction to occur after illumination stops, determined by the longest kinetic components of the recombination in TiO_2_. This is on the order of ~70 ms [13]. With LED light sources, it could be feasible to examine a pulsing time below this limit, but the present system needed only minute-scale on-off sequences. A potentially practical application of a reduced recombination storing charge is shown, an increase in the light energy efficiency by optimizing the pulse widths and duty cycle in pulsed illumination. Of course there is a trade off in longer the overall residence time required to receive the dose.

Dark *E. coli* inhibition suggests that a system like TiO_2_/WO_3_ might overcome the limitation on a recycling design of a solar reactor for water disinfection plants, since the potential for organism regrowth in the dark phase of the recycle operation has been considered a barrier to the use of recycling.

#### 2.4.3. Carbon

Carbon presents the problem of a co-catalyst that may play either of the two roles or both. For example, Torimoto *et al*. [1] included activated carbon as an effective adduct to TiO_2_ among a list of adsorbents. In contrast, Yao *et al*. [12] report an elegant series of experiments with composites of carbon nanotubes and nano-TiO_2_ that are interpreted entirely in terms of electron transfer to carbon. The transfer is verified by photoluminescence quenching. Twenty to one ratios of TiO_2_ to SWCNTs gave their largest rate enhancement. The argument for electron transfer (*vs*. A/S) is further strengthened by observations on physical mixtures of C with TiO_2_. However, only solution concentrations of the phenol substrate were reported, and surprisingly, measurements of remaining TOC after 4 h showed that P25 alone achieved the greatest carbon reduction (~80%). The most effective catalyst of phenol loss (>80% degradation in one hour) reduced TOC by only about ten percent in 4 h. The long lifetimes of intermediates suggests that A/S may have played a role. Data for surface residuals for [11] experiments would help to clarify.

If carbon nanotubes are good electron acceptors, why are efforts to measure energy storage more limited than TiO_2_/WO_3_? It was noted that W(V) can be stabilized by the capture of a cation and that the hole site reacts with water to provide a compensating H^+^. In contrast, carbon is perhaps the better conductor to pass the electron on to, e.g., O_2_. Good energy storage systems need a good way to stabilize the separated charges.

Here, experiments with two forms of carbon modeled after the literature, the common adsorbent activated carbon [1] and single-walled carbon nanotubes [12], are reported. Carbon has been reported to affect the band gap and absorbance limit of TiO_2_. Irradiation with 365-nm LEDs assures comparison of the same energetic preparation of the excited state of TiO_2_, which helps to focus on A/S *versus* charge transfer. The chosen substrate, SMX, is an agricultural antibiotic that is efficiently adsorbed by carbon, giving both pathways an opportunity. The results are mixed and suggest that both are involved. However, the pulse results imply that electron transfer must be accompanied by efficient transfer to oxygen. This then implicates the superoxide ion produced as a key player in the pathway.

## 3. Materials and Methods

### 3.1. Materials

TiO_2_ (Degussa P25) powder (50 m^2^/g; 15%–30% rutile + 85%–70% anatase) was purchased from Degussa. Zeolite ZSM-5 (400 m^2^/g; Si/Al = 280) was purchased from Zeolyst International and calcined at 500 °C for an hour. Silica gel with particle sizes of 0.2–0.5 mm was purchased from Acros Organic. Single-walled carbon nanotubes (SWCNTs) and powdered activated carbon (AC) were obtained from Sigma-Aldrich and used as obtained without further purification. Sulfamethoxazole (SMX), 2,4,6-trichlorophenol (2,4,6-TCP), atrazine and 4-chlorophenol (4-CP) were selected as model substrates for organic pollutants for this report. They were purchased from Sigma-Aldrich and used as received. High performance liquid chromatography (HPLC)-grade acetonitrile and HPLC-grade water were used as a mobile phase in HPLC. Methyl alcohol, ACS grade, was purchased from Sigma-Aldrich. All of the solutions were prepared using “deionized” water (DI) from a Milli-Q system and characterized by its resistivity (18.2 MΩ).

#### 3.1.1. Synthesis of ZSM-5 Containing Catalyst

A three-component (TiO_2_, ZSM-5 and silica gel) composite photocatalyst was prepared as follows. First, ZSM-5 and TiO_2_ were separately dispersed in 20 mL of methanol and sonicated for 30 min. Then, the ZSM-5 suspension was added to the TiO_2_ suspension during stirring, and the ZSM-5/TiO_2_suspension was stirred for 15 min. This was followed by adding the silica gel powder during stirring, which continued for 15 more min. Finally, the methanol was evaporated while stirring; the composite catalyst was dried in an oven at 100 °C. then calcined in a furnace at 500 °C for 3 h. The composite catalyst components weights were maintained to obtain a ratio of TiO_2_:ZSM-5:silica gel = 0.3:0.5:0.5 in the finished form of the catalyst. Above, PZS is used to refer to this ZSM-5-containing composite catalyst.

#### 3.1.2. Synthesis of TiO_2_/WO_3_ Composite

WO_3_/TiO_2_ composite was prepared based on a sol-gel method [12] with titanium isopropoxide and phosphotungstic acid (H_3_PW_12_O_40_) as precursors. Titanium isopropoxide (3.0 mL) and a specific amount of H_3_PW_12_O_40_ were dissolved in 20 mL of isopropyl alcohol and deionized water, respectively. The alcohol solution was then added dropwise to the aqueous solution. After aging for 2 h, the white gel formed was dried at 100 °C and sintered at 500 °C for 5 h. The crystalline structure of this photocatalyst is reported to be anatase with an average particle size of 9.5–10 nm and a molar ratio of 0.04 for W/Ti [12].

#### 3.1.3. Synthesis of Carbon/TiO_2_ Composites

TiO_2_/SWCNTs and TiO_2_/AC composites with different mass ratios were prepared by a simple evaporation and drying process according to Yao *et al*. [12]. First, AC or SWCNTs were dispersed in 100 mL of water and sonicated for 10 min. TiO_2_ powder was added to the suspension and sonicated for 20 more minutes. Then, the suspension containing AC or CNTs and TiO_2_ particles was heated to 80 °C while stirring with air flowing across the suspension’s surface to accelerate the evaporation of water. After the water evaporated, the composite was dried overnight in an oven at 104 °C. SWCNT composites were characterized by SEM and EDX. Carbon is found to be non-homogeneously distributed over the TiO_2_. EDX measurements show that SWCNTs penetrate into the space between individual crystallites in the commercial TiO_2_ aggregates.

### 3.2. Adsorption Isotherms

Adsorption studies were carried out in a batch mode as follows. Accurately weighed amounts of catalysts were added separately into glass vials containing an exact volume (20.0 mL) of different known initial concentrations of SMX. The glass vials were stirred in the dark for 45 min; then, the samples were filtered with a 0.20-μm syringe filter, and the residual concentration of SMX was measured. The amount of adsorbate uptake capacity at equilibrium, *q_e_*, was calculated by mass balance as follows:

$$q_{e}=\frac{V\left(C_{i}-C_{e}\right)}{m}$$

where *q_e_* is the equilibrium amount of solute adsorbed per unit mass of adsorbent (mg/g) and *C_i_* and *C_e_* are the initial and the equilibrium concentrations of the solute (mmol/L) in solution, respectively. V is the volume of the solution, and m is the weight of the adsorbent (g).

### 3.3. Photocatalytic Procedures

In all cases, the reactivity of the photocatalysts was measured as the disappearance of the substrate compounds (SMX, 2,4,6-TCP 4-Cl-phenol and atrazine). Accurately weighed amounts of different prepared photocatalyst composites were dispersed separately into a glass vial containing an exact volume (20.0 mL) of known initial concentration of the model substrates in a single component system. After 30 min of dark stirring, the suspension was irradiated. The reaction vessels were placed in a circular bench-scale 365-nm LED photoreactor, locally fabricated [14]. The inside diameter and depth of the reactor are 9 and 7 cm, respectively, and it is equipped with 90 LED 3-mW output lamps (NSHU5518), which are evenly distributed in 15 rows. The light intensity in the vessel was (4.3 ± 0.2) × 10^16^ photons/s. A sample was withdrawn at pre-determined time intervals, centrifuged, filtered using a 0.2-μm syringe filter, and the residual concentration was measured by HPLC. Some surface concentrations were estimated from isotherms. In selected cases, compounds on the surface of composites and Degussa P25 were extracted with acetonitrile, centrifuged, filtered and the concentration measured by HPLC. Samples for total organic carbon (TOC) (irradiated for one to two hours) were centrifuged, passed through a 0.45-μm syringe filter and analyzed using an Apollo 9000 combustion TOC analyzer equipped with an autosampler.

### 3.4. Analytical Procedures

HPLC: A “Varian pro star 210” HPLC equipped with a PFP 100A column (Phenomenex kinetix^TM^ 2.6 µm, LC Column 100 × 4.6 mm**)** with 20-μL injections and a 325 LC UV-Vis detector was used for the analysis of several substrates. Isocratic elution with a solvent mixture of 50% acetonitrile and 50% water at a flow rate of 1.00 mL·min^−1^ was used for the analysis of SMX. For phenols, a solvent mixture of 50% acetonitrile (0.1 formic) and 50% water (0.1 formic) at a flow rate of 1.25 mL·min^−1^ was used, and for atrazine a solvent mixture of 65% acetonitrile (0.1 formic) and 35% water (0.1 formic) at a flow rate of 1.00 mL·min^−1^ was used. The wavelength of detection was 270, 254 and 220 nm for SMX, phenols and atrazine, respectively. Coliform estimation: Total coliforms and *E. coli* counts were estimated by the Colilert tray method, which is widely employed in water monitoring. This process used trays and procedures from IDEXX Corporation for the Quanti Tray/2000^®^ system. The most probable numbers (MPN) of colonies were estimated with the IDEXX MNP estimator.

### 3.5. Pulse and Stored Electron Studies

A series of experiments were performed to investigate the effect of the pulsed illumination. A locally fabricated programmable controller was used if a light pulse frequency from 1 to 999 ms was required; otherwise, manual off/on was used to achieve pulsing. For fast pulsing, illumination (on) and dark (off) periods in the 200–990-ms range and duty cycles of 10, 30 and 50% were used.

For stored electron measurements, a sample of 0.02 g (WO_3_/TiO_2_) plus 2 mL of 33 ppm 4-chlorophenol (4-CP) was irradiated for a chosen time. At that time, 2 mL iron(III) perchlorate (1 × 10^−3^ M) is added. Then, 1.0 mL to 1.0 mL acetate/acetic acid buffer (pH = 5.5), 0.5 mL ammonium fluoride (0.1 M) plus 0.5 mL 1,10-phenanthroline (0.1 M) are added. The number of electrons stored is calculated based on the absorbance of iron(II) phenanthroline at 510 nm [11].

## 4. Conclusions

The purpose of this study was to explore the relationship of “A/S” and “electron transfer” mechanisms to improve the efficiency of the degradation of organic compounds using three classes of photocatalysts: zeolite-TiO_2_, WO_3_-TiO_2_ and carbon-TiO_2_. The choice was dictated by the aim of comparing two cases, where only one of the mechanisms could be operative, to a case where both might compete. The specific mechanism for the zeolite is A/S, where WO_3_-TiO_2_ has identified electron transfer with little difference in surface properties between the two components of the composite. Both A/S and electron transfer can contribute to carbon-TiO_2_ systems.

The A/S mechanism must balance adsorption with surface mobility to complete substrate delivery to TiO_2_. When these two balance, gains can be disappointing, but the present results also show that it is important to monitor surface concentrations to avoid misleading evidence of “enhancement” from solution-only data.

Electron transfer has been confirmed to be contributed to by carbon. In the case of the carbon forms tested here, the gains are minimal. Only small improvement was observed in the case of SWCNTs, and the effect of carbon was detrimental in the case of using AC. This indicates slow surface diffusions, which can even overcome the effect of electron transfer and reduced recombination. However, some degree of limitation may reflect the absence of a relatively stable site for the transferred electron. Pulse experiments indicated short electron storage lifetimes. The long storage lifetime in WO_3_ illustrates the desired condition, a stabilized electron site analogous to W(V).
